# Design of Brand Business Model Based on Big Data and Internet of Things Technology Application

**DOI:** 10.1155/2022/9189805

**Published:** 2022-09-08

**Authors:** Sheng Yao

**Affiliations:** Guangdong Fanshi Tuoying Brand Planning Co Ltd, Guangzhou 510640, Guangdong, China

## Abstract

Appropriate business models are very important for enterprises. With the advent of the new century, the rapid development and application of technologies such as the Internet of Things and big data have become more and more widely used, which provides great convenience for people's daily life. With the rise and development of the Internet of Things and big data technology, human work and life have become easier and easier, and it has also greatly changed human production and development. Under the fierce market competition, enterprises pay more and more attention to the independent research and development capabilities of their own brand products. With the rapid development of the Internet age, the status of brands in the improvement of business models is also increasing. The brand business model design must not only meet the ever-changing market consumer demand but also conform to the ever-changing fashion trends. The article briefly analyzed the development and application of the Internet of Things and big data technology and optimized the design method of the brand business model by using big data technology in design of the brand business model. On this basis, a decision support method for a brand business model based on a data warehouse was proposed to achieve scientific, efficient, and accurate brand product development. In order to understand consumers' consumption habits and awareness of brands, the article randomly selected 400 people to conduct a survey. It was found that 45% of male respondents preferred commercials with simple and elegant colors; 38% of female respondents preferred commercials with minimalist styles. Modern consumers are mainly pragmatic, and the design of brand business models should be developed in this regard.

## 1. Introduction

With the advent of the era of big data, human society has entered the digital era. The massive increase and penetration of data and information have driven tremendous changes in people's living and production levels, as well as changes in people's consumption needs. It is driven by the continuous development and progress of high-tech such as information technology, network technology, and social media technology, business trends are rapidly changing and changing, presenting a variety of popular information. The intersection of computer networks, e-commerce, information technology, and other fields has resulted in an ever-increasing amount of massive data. In this case, as soon as the concept of big data appeared, it has received extensive attention from scholars, experts, and managers from all over the world. The reason why big data is “big” is not because it is too numerous, but because it has too many types, such as web page scanning, location information services, online and offline consumption, and so on. Under the background of fierce market competition and economic globalization and competition integration, localization, and homogeneity, more and more enterprises pay attention to corporate image, strengthen the core competitiveness, and improve brand awareness. At the same time, they also recognizes the importance of design to the enterprise.

Economic globalization has made it possible for companies from all countries to participate in world competitions and share the world's advanced development achievements. At this crossroads, which is both an opportunity and a challenge, almost all companies have thought of the importance of brands. Lee and Soon's purpose was to explore the Apple iPhone jailbreak phenomenon, a new scenario in which a company actively suppresses and discourages loyal consumers from cocreating value and customizing its products. Lee and Soon provided a comprehensive conceptual model to show how different theories of consumer behavior are synthesized in this new phenomenon. Compared to previous work, consumer activists were committed to the brands and products they boycott. Instead of switching to other brands, these jailbreakers and hackers stay loyal to the product and really try to help the brand [1]. Although there is a lot of research on Perceived Brand Globalization (PBG), the concept itself is far from clear and more in-depth research is needed. Mandler aimed to contribute to the global brand literature by proposing a scalable PBG concept and examining the impact of traditional versus global brands in practice. Clearly, the impact of international brands was not all positive. The result provided a clearer perspective on the rise and fall of managers' awareness of brand globalization and called for future research on global brands to be included in all levels beyond the brand, so as to fully grasp this phenomenon, but it's not very practical [2]. Huaman-Ramirez et al. aimed to expand the comprehension of how digital platforms actively impact brand trust by presenting two new variables mediated by brand affection and brand creativity, and by testing the modifying role of consumer e-centrism in these associations. Research had shown that brand globalization was positively correlated with brand sentiment, which in turn was positively correlated with brand trust, which suggested a weaker relationship between brand globalization and brand sentiment for ethnocentric consumers. Brand globalization was positively correlated with brand innovation, which in turn was positively correlated with brand trust, which was a weaker relationship between brand globalization and brand innovation for ethnocentric consumers. This research presented new opportunities for managers concerned with optimizing global brand management. First, the findings suggested that managers were interested in increasing the emotional and emotional aspects of their global brands, making them more trustworthy. Secondly, it was important for brand executives to also highlight the innovative sides of their global brands. Indeed, it was important for practitioners not only to constantly come up with new and exciting propositions for citizens but as well as to keep up with the recent trends in the market. The better managers offered new and helpful resolutions to meet consumer needs, the more consumers would trust these global brands [3]. The results of the study indicated that Sarkar et al. aims to find previous research work on brand cocreation and the role of different stakeholders in brand cocreation and to propose a conceptual tool that can be empirically validated. The results of the study showed that the history of literature presented two main stakeholders, namely, consumers and organizations. The importance of the third important stakeholder, the supplier, had been mostly ignored. However, previous studies had shown that properly managed supplier involvement could reduce the time and cost of product and brand development, and it could provide newer technologies and better quality. Therefore, the importance of suppliers for the creation of a successful brand together cannot be forgotten. In addition to studying the incentives and results of brand cocreation from the standpoint of consumers and organizations, the aim was to examine the supplier's point of view of the brand cocreation framework. This was the first review of the scholarly literature on brand cocreation from the point of view of three key stakeholders: consumers, organizations, and suppliers [4].

The circular economy (CE) is gaining significance, partly due to the rarity of existing natural assets useable in the context of the environment and changes in collective consumer actions. Cutting-edge technologies such as Big Data and the Internet of Things (IoT) have the possibility to pry the concept of CE from being introduced in organizations and societies and becoming more present in everyday life. Nobre and Tavares conducted a content and social network analysis by using the “R” statistical tool and then made comparisons with a number of current industry initiatives. The results showed that China and the United States were the countries most interested in this area and revealed a context with significant research opportunities [5]. Advances in wireless technologies, computing, and sensing devices, as well as the decreasing cost of these technologies, were driving and accelerating the development of cyber-physical systems. These systems used the IoT model to provide various types of services, such as monitoring, weather monitoring, vehicle traffic management, and controls of production activities. The development and adoption of these systems still face various challenges. On the one hand, the development challenges are primarily related to security, robustness, availability, adequate performance, and optimization of energy savings. On the other hand, the use of these systems generates large amounts of fine-grained data that need to be processed and correlated, commonly through big data analyses to extract useful knowledge that can be used by the software services that control them. Ochoa et al. presented a novel contribution to the design, implementation, and use of these systems as part of a special issue on cyber-physical systems, the Internet of Things, and big data [6]. In addition to the functions of self-monitoring, self-diagnosis, and self-protection, intelligent electrical equipment also has the functions of digitally monitoring electrical parameters, implementing judgments, and intelligently controlling and protecting the monitored objects. Jianhua et al. believed that it was reasonable to analyze and judge the status of in-service power equipment, and to use the method of life cycle management for predictive maintenance, thereby improving the reliability and safety of the entire electrical system. It has become an irreversible trend to use big data-related technologies to process the online monitoring parameters of power equipment, so as to realize the full life cycle management of power equipment. In the future, power equipment manufacturers should provide comprehensive and in-depth services to their customers, that is, become service providers, not just physical products of power equipment. The operating parameters of power equipment are obtained through the Internet of Things, and their life status is analyzed based on big data technology [7]. These studies provided a detailed analysis of brand design and IoT big data technologies. It was undeniable that these studies had greatly contributed to the development of the corresponding fields, and a lot of experience could be learned from methods and data analysis. However, the research on brand business model design based on big data and Internet of Things technology was relatively few and not thorough enough, and it was necessary to fully apply these technologies to the research in this field.

In the research of commercial brand design combined with big data and Internet of Things technology, it was found that the business model of the e-commerce platform has developed by leaps and bounds. Among them, the transaction scale structure of the B2C sales model had increased year by year, accounting for 64% in 2018. In the survey of consumers' consumption habits and brand awareness, it was found that 82% of consumers liked to travel and other outdoor activities and more than 75% of consumers were used to buying goods at special counters in department stores or shopping malls, and about 40% of consumers preferred brand commercials with simple colors and simple style.

## 2. Design Method of Brand Business Model Based on Big Data and Internet of Things Technology Application

The business model is to combine internal and external factors to form an efficient operation system with unique core competitiveness, so as to achieve the best interests of customers. The rapid development of business models has an important relationship with the advancement of technology, the rise of the Internet, and the rise of e-commerce [8]. The speed and tolerance of consumers accepting new things make many companies adjust their corporate and brand strategies at any time, trying to continuously meet the sales hot spots in the market through efficient design and operation of management activities.

The early marketing methods of enterprises were relatively simple. Generally, enterprises made a series of marketing plans through market research, and finally made decisions and sold products through written data. The whole process was one-way. This one-way information research method had no practical significance to the marketing strategy of enterprises. Enterprises still cannot have a deep understanding of customers, and there was no way to connect the relationship between enterprises and customers, so they could not provide better services for customers [9]. Although some companies further used the method of telephone return visits to customers to improve customers' loyalty to the brand, this kind of telephone consultation service method was very unpopular. This method was not only extremely inefficient but also unsatisfactory [10]. After that, many companies also discovered the limitations of the telephone return visit method, so some companies pushed the promotion activities of the merchants through SMS to attract customers. However, the time for this passive marketing method to benefit was very short. On the one hand, customers were easily numb or even annoyed by this behavior. On the other hand, because there was no way for enterprises to screen according to the needs of customers, and could not convey effective information to customers who really need it, the effectiveness of the marketing method of SMS push was obvious [11].

**Figure 1:**
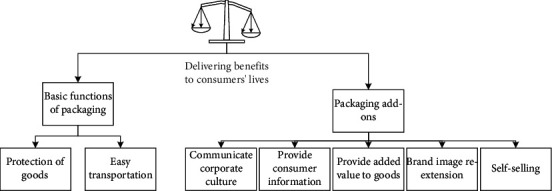
Requirements for brand design in the existing consumer market.

With the development of science and technology, the Internet has gradually become known to people. By building official brand websites, companies allow customers to understand corporate and brand information more quickly. Enterprises have established the most direct contact information with customers. Customers can find out about enterprises at any time by searching for enterprises and brands on the Internet platform, and can quickly obtain the contact information of enterprises. Although this method of network construction can let people know about enterprises and brands in a faster and more convenient way, this method of publicity is still single information dissemination, and enterprises are still unable to actively provide timely services to customers in need until the mobile internet appeared [12]. If an enterprise does not have Internet thinking and does not know how to apply the large platform of the Internet, it cannot become bigger and stronger if it is limited to traditional store sales. Under the background of the popularity and history of today's mobile Internet, enterprises not only have to have Internet thinking to reasonably apply network platforms for strategic marketing but also need to follow the current trend of technology—WeChat marketing thinking [13]. As an indispensable communication method in people's daily life, WeChat is used as an important means of information acquisition, and WeChat brings customers together. Through WeChat, a well-known communication platform, the communication method between the enterprise and the customer is close to zero distance like relatives or friends. By following the company's WeChat public account, customers can learn about the company's dynamics and brand culture, and obtain information on the latest research and development products and activities. And enterprises can manage more effectively for different customer groups by establishing a complete customer information database. This not only facilitates timely communication and effective interaction between enterprises and customers but also pushes the latest product and activity information developed by the company to customers. It can also answer all customer questions and receive feedback and suggestions from customers through intelligent replies and artificial real-time [14]. Many commodities, like the previous products, are aimed at primary consumers. They are directly sold to the market without packaging and have no inherent brand image. Most of the target groups are grassroots working people as the main consumers. With the rapid development of the Internet industry and the continuous expansion of the market, the positioning of the original commodities makes them face the bottleneck of sales, which increases the unnecessary waste of most commodities [15]. The current sales situation is gradually declining. In other words, in order to change the current low development situation, it is necessary to change the positioning of the products. It is necessary to ensure the intimacy of products from nature, and it is necessary to meet the sales concept that products come from pure nature without additives, so as to meet people's emotional and psychological needs, make the product itself have a human touch, and stimulate consumers' desire to buy. It is also necessary to build people's loyalty to the brand [16]. Brands not only represent the main products and services in the exchange process but also reflect consumers' deepening psychological feelings and acceptance of commodity value. However, with the changes in the consumption behavior of consumer groups and the turning of their habits, in the face of the rapid changes in people's consumption behavior, some well-known brands with a long history of precipitation cannot fully meet the needs of all consumers. Therefore, the products of the e-commerce platform are resegmented again, and the target market positioning is refined again. The repositioning of consumer groups makes the brand younger, and establishes an internal connection between the brand and this normal consumer group, as shown in Figure 1 [17].

**Figure 2:**
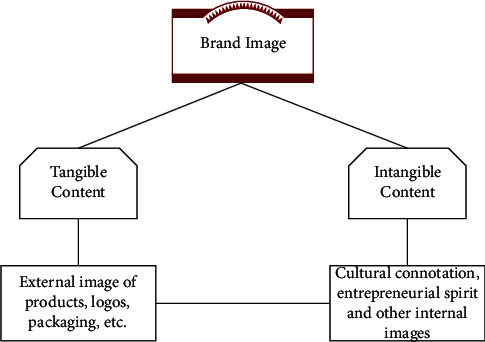
Brand image content.

In today's era, the brand not only represents the image of an enterprise, but also a symbol of the enterprise's management strength and innovation ability. The number of world-famous brands also symbolizes the strength of the country's comprehensive competitiveness. The material composition of the value of the brand business model is mainly reflected in the technical level of the product, the design quality of the product, and the manufacturing quality of the product. One of the key points involved in the brand concept is the establishment of brand image, which is based on the perception subject—consumer usage and perception experience—and then it forms the psychological association to the brand. In terms of expression, it includes two aspects (1) Tangible content (external): the functional content of the brand, which is the functional needs of consumers that the products and services of the brand can satisfy; that is, the external intuitive features of products, logos, packaging, etc., which can be directly contacted and seen by consumers, and the intuitive feeling of brand image on the basis of material [18]; (2) Intangible content (intrinsic): the charming characteristics of the brand, that is, the personality characteristics that the entrepreneur endows the brand and is perceived by consumers. It usually includes the brand's cultural connotation, entrepreneurial spirit, and other internal characteristics, which are the key factors to determine the success of the brand [19], as shown in Figure 2.

**Figure 3:**
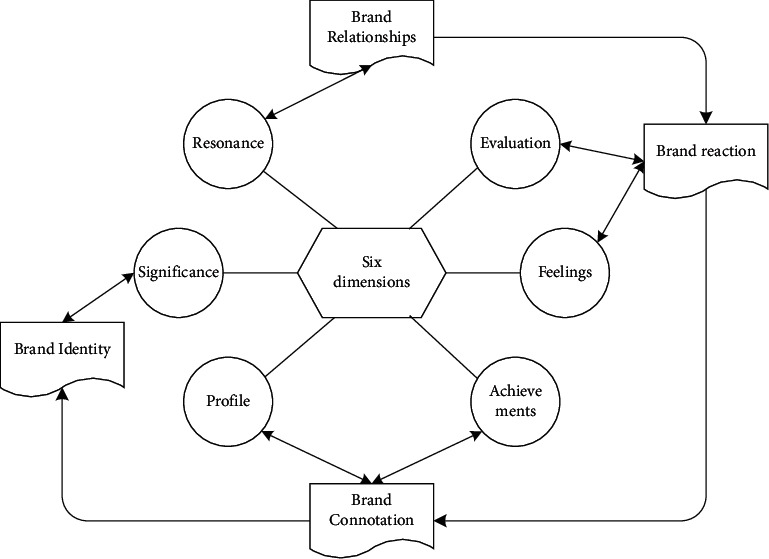
Customer value model based on brand equity.

The derivation of brand value is nothing more than accompanying the growth of customer demand. The promotion of brand value and the creation of customers are inseparable and complementary, and they together constitute the cornerstone of the sustainable development of enterprises [20]. Generally speaking, brand value is mainly composed of brand premium, satisfaction, loyalty, perceived quality, leadership, perceived value, brand personality, organizational association, brand recognition, market share, price, distribution index, etc. [21]. Almost most of them are customer value-oriented factors, so a customer value model based on brand equity is used to illustrate it vividly, as shown in Figure 3. And brand maintenance is particularly important as an inherent requirement for corporate image and large-scale long-term development. Brand maintenance includes standardized management and testing of product quality, operating technology, and related equipment. More importantly, it is necessary to continuously improve and implement corporate culture and promote the overall improvement of corporate management.

**Figure 4:**
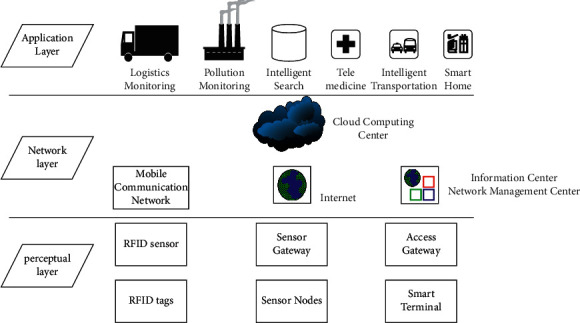
Schematic diagram of IoT architecture.

Internet of Things (IoT) is a system that connects people to people, things to things, and people to things for information exchange through various proprietary networks, such as communication networks, sensor networks, and industry-specific networks. Currently, with the development of an increasing number of sensing technologies, such as infrared and 4G, IoT is defined as a network where all objects are connected to the Internet for communication through sensing devices. The current IoT architecture is usually considered to contain three layers: sensing layer, network layer, and application layer, as shown in Figure 4.

**Figure 5:**
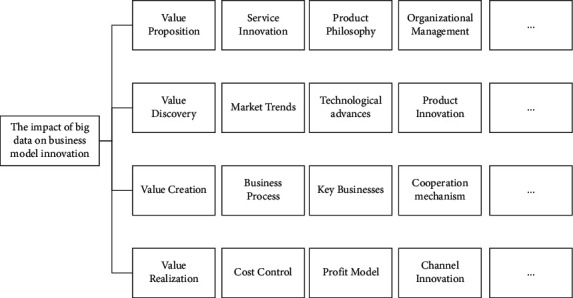
Model of the impact of big data on business model innovation.

With the development of science and technology, people have entered the era of “big data,” and big data is becoming the core competitiveness of business competition in this era. The generation, development, and application of big data pose challenges to traditional business models and provide opportunities for continuous innovation of business models. From the previous summary and analysis, it can be seen that big data has an important impact on the content of the four elements of business model innovation. The realization of business model innovation or transformation from the perspective of these four elements is an important condition for enterprises to realize business model innovation. Therefore, this paper constructed a model of the impact of big data on business model innovation, as shown in Figure 5.

Along with the speedy progress and application of information technology, computer technology, and Internet technology, human society has rapidly moved into a modern digital era. With the development of the network, the continuous development of the Internet, mobile, social networking, and other fields, the application scope of network information technology has been greatly expanded. As a positive factor, “data” is constantly eroding people's lives, and the explosion of information has led to a large amount of data. The continuous increase in the amount of information has led to qualitative changes in people's thinking and behavior, which also heralds the advent of the era of big data.

It is supposed there are *N* customers with *K* brands to choose from, or they can choose not to buy, that is: customer *n* (*n* = 1,2,…,*N*) has *K* + 1 kinds of products to choose from, *k* = 0,1,…,*K*, respectively. For the *i*-th customer facing *K* + 1 choices, it is supposed the utility of choosing k is

(1) $$\begin{array}{c} U_{nk}=X_{n}^{\prime }\beta_{k}+\varepsilon_{nk},n=0,1,\ldots ,K\text{.} \end{array}$$

Among them, *X*_*n*_′ is a vector representing the intrinsic features of the customer, *β*_*k*_ is also a vector representing the parameters for the intrinsic features of the correspondent customer when the *k*-th good is selected, and *ε*_*nk*_ is a random term that is separate and obeys.

(2) $$\begin{array}{c} \varepsilon_{nk}∼N\left(0,1\right)\text{.} \end{array}$$

*p*_*nk*_ is to express the possibility of customer *n* choosing brand *k*. Then based on the principles of utility maximization, the probability of a customer *n* choosing brand *k* can be obtained as follows:

(3) $$\begin{array}{c} p_{nk}=P\left(U_{nk}>U_{nj},j\neq k\right)\text{.} \end{array}$$

The density function for the unobserved utility function is

(4) $$\begin{array}{c} f\left(\varepsilon_{nk}\right)=e^{-\varepsilon_{nk}}e^{-e^{-\varepsilon_{nk}}}\text{.} \end{array}$$

After integration, the cumulative distribution function is obtained as follows:

(5) $$\begin{array}{c} F\left(\varepsilon_{nk}\right)=\exp\;\left(-e^{-\varepsilon_{nk}}\right)\text{.} \end{array}$$

From the random utility part, it can be known that the probability of a customer *n* choosing option *i* is

(6) $$\begin{array}{c} p_{ni}=P\left(C_{ni}+\varepsilon_{ni}>C_{nk}+\varepsilon_{nk},\forall k\neq i\right)=P\left(\varepsilon_{nj}<\varepsilon_{ni}+C_{ni}-C_{nk},\forall k\neq i\right)\text{.} \end{array}$$

If *ε*_*ni*_ is given, this expression is the cumulative distribution function of every *ε*_*nk*_ on *ε*_*ni*_+*C*_*ni*_ − *C*_*nj*_, which can be expressed as exp( exp(*ε*_*ni*_+*C*_*ni*_ − *C*_*nj*_)) by formula (5). Since *ε*_*nj*_ is independent, this cumulative distribution is the result of multiplying a single cumulative distribution for all *k* ≠ i; that is, in the presence of random interference, the conditional probability that customer *n* chooses *i* is as follows:

(7) $$\begin{array}{c} p_{ni}\left|\varepsilon_{ni}\right.=\prod_{k\neq i}e^{-e^{-\left(\varepsilon_{ni}+C_{ni}-C_{nk}\right)}}\text{.} \end{array}$$

Formula (7) is substituted into the distribution to solve *p*_*ni*_

(8) $$\begin{array}{c} p_{ni}=\int \left(p_{ni}\left|\varepsilon_{ni}\right.\right)d\varepsilon_{ni}\text{.} \end{array}$$

It can get

(9) $$\begin{array}{c} p_{ni}=\int_{\varepsilon_{ni}=-\infty }^{+\infty }\exp\;\left(-e^{-\varepsilon_{ni}}\underset{k}{\sum }\exp\;\left(-\left(C_{ni}-C_{nk}\right)\right)\right)e^{{-\varepsilon }_{ni}}d_{\varepsilon_{ni}}t=e^{{-\varepsilon }_{ni}}\text{.} \end{array}$$

The formula (9) is substituted to get

(10) $$\begin{array}{c} p_{ni}=\int_{t=\infty }^{0}\exp\;\left(t\underset{k}{\sum }\exp\;\left(-\left(C_{ni}-C_{nk}\right)\right)\right)dt={\left.\frac{\exp\;\left(t\underset{k}{\sum }\exp\;\left(-\left(C_{ni}-C_{nk}\right)\right)\right)}{\left.\underset{k}{\sum }\exp\text{ (}-\text{(}C_{ni}-C_{nk}\text{)}\right)}\right|}_{\infty }^{0}p_{ni}=\frac{1}{\underset{k}{\sum }e^{-\left(C_{ni}-C_{nk}\right)}}=\frac{e^{C_{ni}}}{\sum_{k}e^{C_{ni}}}\text{.} \end{array}$$

And from formula (1), it can get

(11) $$\begin{array}{c} p_{ni}=\frac{e^{x_{n}^{\prime }\beta_{i}}}{\sum_{k=0}^{K}e^{x_{n}^{\prime }\beta_{i}}}\text{.} \end{array}$$

If *β*_*j*_^*∗*^=*β*_*j*_+*m* is defined for any vector *m*, and then the probabilities defined in formula (11) are recalculated using *β*_*j*_^*∗*^ instead of *β*_*j*_, the result is the same as using *β*_*j*_, because the terms containing *m* are eliminated. That is, adding any customer attribute variable *m* has no effect on the results, so it can be concluded that customer attributes, such as gender and occupation., have no effect on the results. For this purpose, *β*_0_=0 can be normalized. (Because the probabilities sum to 1, only *K* parameter vectors are needed to determine *K* + 1 probabilities) so the probabilities are

(12) $$\begin{array}{c} p_{ni}=\frac{e^{x_{n}^{\prime }\beta_{i}}}{1+\sum_{k=1}^{K}e^{x_{n}^{\prime }\beta_{k}}}\text{.} \end{array}$$

By formula (12), the *K* log odds ratios can be calculated as follows:

(13) $$\begin{array}{c} \ln\left(\frac{p_{ni}}{p_{nk}}\right)=\ln\frac{e^{x_{n}^{\prime }\beta_{i}}/1+\sum_{k=1}^{K}e^{x_{n}^{\prime }\beta_{k}}}{e^{x_{n}^{\prime }\beta_{k}}/1+\sum_{k=1}^{K}e^{x_{n}^{\prime }\beta_{K}}}=x_{n}^{\prime }\left(\beta_{i}-\beta_{j}\right),j\neq i\text{.} \end{array}$$

If the model has *K* + 1 categories, it can be expressed as follows:

(14) $$\begin{array}{c} \ln\left(\frac{p_{n0}}{p_{nK}}\right)=x_{n}^{\prime }\left(\beta_{0}-\beta_{K}\right)\text{,}\\ \ln\left(\frac{p_{n1}}{p_{nK}}\right)=x_{n}^{\prime }\left(\beta_{1}-\beta_{K}\right)\text{,}\\ \ln\left(\frac{p_{n\left(K-1\right)}}{p_{nK}}\right)=x_{n}^{\prime }\left(\beta_{K-1}-\beta_{K}\right)\text{.} \end{array}$$

Among them, *K* is the reference category, that is, *k* is the standard to compare with other options.

In product development, the use of big data resources—mainly consumer input and the objective grasp of popular information—this open and innovative way can enhance the specific operation in the design work and reduce the cost of research and development, so as to achieve the ability to respond quickly to consumer and market changes. At the same time, it also plays a key role in realizing the practicability, science, and value of product design.

Through the theory of value sharing, enterprises, customers, and other stakeholders must establish sufficient connections, maintain continuous interaction, and form effective aggregation, so as to promote the overall value creation of the enterprise system. Based on the brand community, it can be divided into three business models as follows: (1) take the enterprise as the center, strengthen the company's functions, and improve the customer's products and services; (2) taking the customer as the center, create customer experience shaping with a special experience for customers; (3) focus on stakeholders, and build a broad set of values for members of society by strengthening the connection of various stakeholders, as shown in Table 1.

## 3. Experiment Preparation for Brand Business Model Design Based on Big Data and Internet of Things Technology Applications

Successful brands usually have a strong brand identity with concise expressions, diverse forms, and distinct personality traits. A representative brand image will show the essential characteristics of the company and show it in a specific way, allowing customers to have a visual impact, thereby improving the recognition of the product and distinguishing it from other products. As a corporate brand design, there should be distinct differences to facilitate the understanding of customers; at the same time, elements that best reflect the company's characteristics should be incorporated into the design.

**Figure 6:**
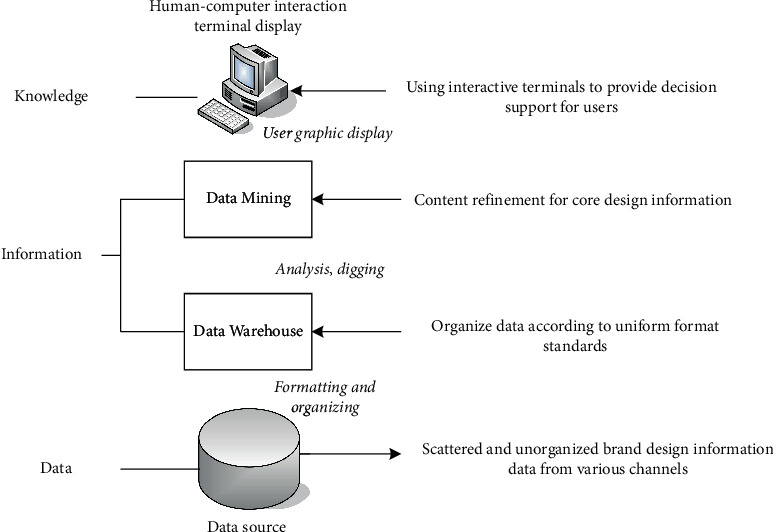
Basic process of brand design decision support system based on a data warehouse.

In today's increasingly abundant information resources, massive information resources will become an important part of the development of human society. The use of information technology to assist product development has become a new development trend. In the process of information technology guiding product design, obtaining consumers' input and contributions through a large number of consumer information resources is an important means to complete product development. Numerous good companies have begun trying to mine and quantify consumer opinions and preferences from traditional data sources, including point-of-sale data and customer feedback. They distill key information to aid product design from new data sources including consumer reviews on social media and third-party platforms, and sensor data describing actual product usage. The decision support method for brand product design based on data warehouse is shown in Figure 6.

At the end of the data collection phase, the company has a complete set of data collection and archiving tools and can either sell the collected data to larger companies that can analyze it, or provide data collection and archiving services to companies without data collection systems. Once data analysis is complete, the company is again fully capable of data extraction and can connect data extraction activities. Once the analysis and statistical reporting are complete, the company can sell the net data mining business.

Once the data application phase is complete, companies are no longer limited to data sales policies. Selling data has no commercial value for the company's core business, but can adjust and design business around the relationship between data, and effectively implement data-driven business.

The terminal is at the end of the supply chain and is the place where consumers have the closest contact with the brand, so the image of the terminal directly affects the image of the brand in the minds of consumers to a certain extent. And the brand is generally independent, there are franchise chains, there are entrusted companies, and the standard franchise image is uniformly designed. Some brands have high franchise fees and need an optimal location. Classic business districts do not allow returns, and these strict conditions cannot be returned. These strict franchise conditions can control the expansion speed of the brand, and also ensure the relevant service quality of the store to a certain extent. In order to understand consumers' consumption habits and awareness of brands, 400 consumers in area A were randomly surveyed. The basic information of the respondents is shown in Tables 2 and 3.

## 4. Brand Business Model Design Data

**Figure 7:**
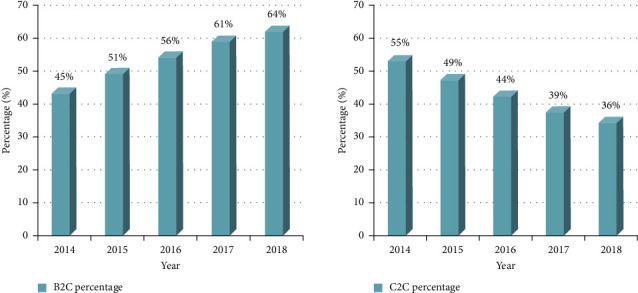
2014–2018 China online shopping market transaction scale structure.

In recent years, the development of e-commerce has been very fast, and the types of advertising campaigns will also change at different times, so many sales models will gradually shift from physical stores to e-commerce platforms. In this way, e-commerce can not only expand development in popular industries but also reduce costs for the business itself. This improves the display of products in the sales process, it will be more efficient to face these channels, and the development of e-commerce will be more efficient. Today, when the current monopoly model is changing, in order to meet the needs of customers, future product sales will move in the direction of “digitization, channelization, and platformization.” For most consumers, what they like is the traditional handmade products they make or the deep cultural heritage they have. The current e-commerce platform sales model has become a major sales method, especially since the B2C transaction scale is increasing every year, and by 2018, it has reached 64%, as shown in Figure 7, this sales model has been affected by favored by many young people. If the development model of the e-commerce platform cannot be well grasped, the sales model of professional brick-and-mortar stores will face difficulties and bottlenecks in development.

**Figure 8:**
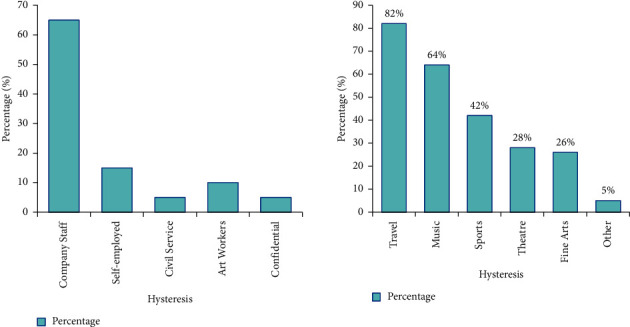
Respondents' occupations and hobbies.

**Figure 9:**
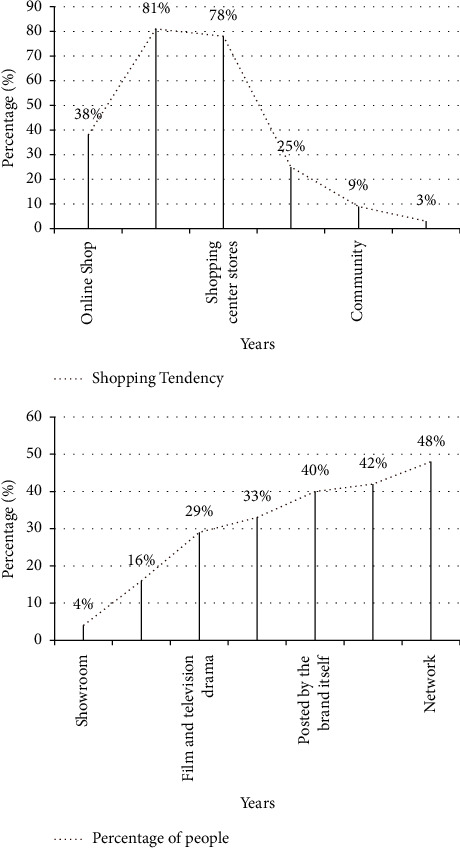
Channels for purchasing goods and channels for obtaining fashion information.

Figures 8 and 9 are the statistical results of the respondents' brand tendencies.

It can be seen from Figure 8 that the highest proportion of respondents' hobbies is travel, accounting for 82%, followed by music 64%, sports 42%, drama 28%, and art 26%.

As can be seen from Figure 9, 81% of consumers are accustomed to buying things on department store counters, and 78% like to buy things in shopping mall specialty stores. More than 40% of consumers shop through trend information provided by the Internet, fashion magazines, and major brands. Other avenues include friend recommendations, movies, outdoor advertising, and fashion shows.

**Figure 10:**
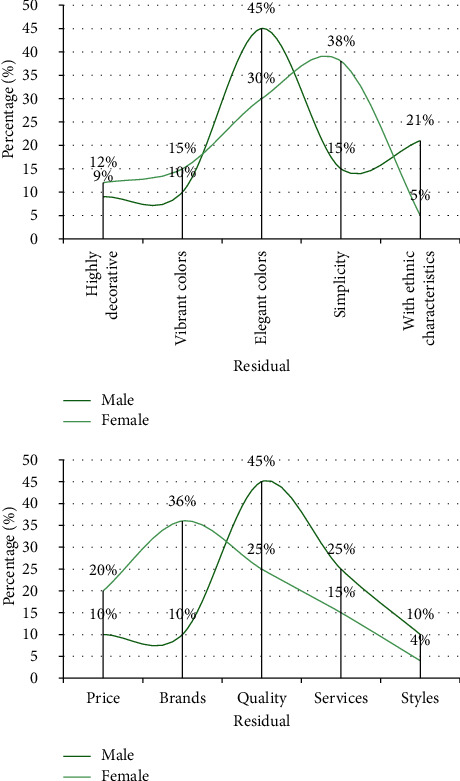
Top aspects consumers pay attention to when purchasing a product.

For the brand business marketing style, there is a large gender difference in the style that the respondents prefer, as shown in Figure 10. The majority of male respondents prefer commercials with simple and elegant colors, accounting for 45%; the majority of female respondents prefer simple-style commercials, accounting for 38%. Regarding the aspects that consumers pay attention to when purchasing products, male customers pay the most attention to the quality of products, and female customers pay the most attention to brands, accounting for 45% and 36%, respectively.

In general, with the development of the times, modern consumers are mainly pragmatic. Consumers have little influence on the company's culture, service concept, store design and layout, and the influence of people around them, as well as advertisements and celebrity endorsements.

## 5. Conclusions

In today's 21st century, with the continuous development of technologies and applications such as computer networks, mobile Internet, and social networks, computer technology is gradually integrated into people's daily lives, and “data” is also constantly infiltrating people's lives. The promotion of brand value is of great significance to enterprises. On this basis, a decision support system for brand design based on a data warehouse was proposed. Data mining technology was used to obtain design element information, decision support for brand design was provided through human-computer interaction tools, and information sharing, communication, and application were realized. The system not only provided an objective and scientific thinking method and theory for brand design but also filled the gap of research theory using information technology theory to assist brand design work. It organically integrated various information technologies and brand design theories to form an efficient, scientific, and accurate brand design method, and refined design information from the brand design information data from multichannel sources. In the era of big data, a brand design must conduct in-depth research on the social environment, transform design thinking, and use information technology to adapt to current consumption and trends.

## Figures and Tables

**Table 1:** Types of business model innovations based on brand communities.

Name	Objectives	Functions	Community characteristics
Corporate function enhancement	Strengthen the business	Enhance corporate functions	Company-led community result-oriented

Customer experience shaping	Strengthen customers	Focus on a lifestyle theme and unique lifestyle	The natural integration of brand value proposition and lifestyle brand community and corporate business activities are moderately divided by customer-led brand community

Cross-border platform integration	Strengthen stakeholder connections	Stakeholders form connections to generate value	Enterprise has brand appeal

**Table 2:** Basic information of respondents.

Test items		Number of people	Proportion (%)
Age	18–25	69	17.25
	26–35	136	34
	35–45	156	39
	45–60	19	4.75
	> 60	20	5.0

**Table 3:** Income of respondents.

Test items		Number of people	Proportion (%)
Gender	Male	200	50
	Female	200	40

Monthly income range	2000–3500	50	12.5
	3501–5000	52	13.0
	5001–8000	230	57.5
	8001–12000	46	11.5
	> 12000	22	5.5

## Data Availability

The data that support the findings of this study are available from the corresponding author upon reasonable request.
